# Widely Targeted UHPLC-MS/MS Metabolomic Analysis on the Chemical Variation in Blueberry-Filled Pastries During Processing

**DOI:** 10.3389/fnut.2020.569172

**Published:** 2020-11-09

**Authors:** Jie Zheng, Zhongjun Wu, Nan Yang, Kangning Zhou, Wenzhong Hu, Shiyi Ou, Pengzhan Liu

**Affiliations:** ^1^Department of Food Science and Engineering, Jinan University, Guangzhou, China; ^2^College of Life Science, Dalian Minzu University, Dalian, China; ^3^Key Laboratory of Biotechnology and Bioresources Utilization, Dalian Minzu University, Dalian, China; ^4^School of Food Science and Engineering, South China University of Technology, Guangzhou, China

**Keywords:** baking, stir-frying, metabolomic analysis, chemical variation, degradation

## Abstract

The majority of components in fruits are sensitive to heat-processing. Nevertheless, fruits are becoming popular ingredients in processed foods, like bakery foods. Therefore, the fate of the components in the fruit-involved food during thermal processing is important for the assessment of their nutritional values and sensory properties. Unfortunately, comprehensive knowledge of the compositional alteration in real food products during processing is limited. In the current study, a popular bakery food, blueberry-filled pastry, was taken as the object, and a widely targeted metabolomic approach was applied to investigate the holistic compositional variation of blueberry filling during pastry preparation. Amongst the total of 630 chemicals identified, 288 chemicals were screened as differential compounds between samples collected at different processing stages. The most variation of the chemicals was observed during the process of stir-frying. A total of 197 chemicals varied significantly in concentrations during stir-frying, while only 75 chemicals altered significantly in contents during baking. Amongst 288 differential compounds, 117 belonged to the group of phenolic compounds, with the others found to be sugars and organic acids, amino acids, lipids, nucleotides, etc. The possible mechanisms of the chemical alterations during thermal processing were also discussed in the current study. The data provide comprehensive information on the compositional changes in berry-containing fillings during thermal processing, and hints and emphasis for further investigation of the underlying mechanisms.

## Introduction

Blueberries contain various phytochemicals with potential health effects, including anthocyanins, flavonols, vitamins, lutein, sugars, and organic acid (1–3). The consumption of polyphenol-rich blueberries and their polyphenol extracts is associated with the protection and management of non-communicable diseases such as cancers, cardiovascular diseases, diabetes, osteoporosis, and neurodegenerative diseases (1). However, the perishable nature and seasonal availability of berries limit the all-year-round supply and consumption. Food processing allows the all-year-round provision and provides various types of blueberry products to compensate for the different requirements and appreciations by the customers. One large group of products are bakery food products filled with blueberries or blueberry containing materials, such as pies, pastries, muffins, cakes, and cookies. Such food products are becoming increasingly popular and are consumed daily in many areas of the world. Processing can prolong the shelf life of blueberry products and increase the diversity of products for customers' choice; however, nutritional compounds, such as polyphenols, may be destroyed to some extent during processing, especially during thermal processing, which in turn affects the health-promoting properties of the products (4–6).

However, knowledge of the effect of thermal processing on the phytochemical profile of the products is limited to a few compounds due to the conventional analytical methods focusing only on a certain group of chemicals (3, 7, 8), although the biological importance is determined by the whole phytochemical profile. Thus, a technique should be developed to fully elucidate the components in a food product for evaluating the nutritional changes during thermal processing. Metabolomic analysis is used to provide a comprehensive chemical profile of as many small molecules as possible in a system (plant, cell, or food material), which allows for a more thorough and encompassing analysis of molecular composition than traditional methods. The application of metabolomic study on the tracking of dynamic compositional changes during food processing could provide a holistic view on the chemical changes and correlated reactions under various treatments of food. Thus, in recent years, metabolomic analysis has been applied in food products, such as in the investigation of nectar beverage of black raspberries (9), roasted coffee beans (10), wines (11), and extra-virgin olive oil (12).

In our previous study (8), the variation in individual flavonoids, including flavonol glycosides and anthocyanins, was investigated in two thermal processing procedures of blueberry-filled pastries. The results showed that stir-frying largely decreased the contents of flavonoids, especially anthocyanins, in blueberry filling, and baking caused less variation in flavonoid profiles. However, the changes in other phenolic compounds, which may be related to the degradation of flavonoids, and the components aside from polyphenols in the fillings remain largely undiscovered. The chemical changes and the underlying mechanisms during food production should be further studied. Food matrix is such a complex mixture that phenolic compounds, with their chemical reactivity, may undergo reactions with various compounds other than their own degradation. Therefore, a holistic view on the variation of phytochemicals in blueberries during processing is important to provide insights into the changes in bioactive compounds, and encourage and give hints for scientists to further evaluate the underlying chemical reaction mechanisms of key varying compounds. Such information on the effect of thermal processing on the phytochemical profiles in real food systems, such as blueberry pastry as in this case, remains largely limited.

The current study aims to provide useful information on the chemical changes in blueberry-containing products during thermal processing by using a widely targeted metabolomic approach. The results will present clues on the degradation of thermal-sensitive components and the interaction between bioactive compounds of the products during processing. The work also provides further insights into the utilization of widely targeted metabolomic techniques in the screening of key chemical changes and the revelation of underlying chemical mechanisms.

## Materials and Methods

### Materials and Chemicals

Fresh blueberries (*Vaccinium* spp.) were imported from Chile, and all the other ingredients of blueberry-filled pastries were purchased from the local market. Methanol, acetonitrile, and acetic acid were of HPLC grade and purchased from Merck (Darmstadt, Hesse, Germany). Double deionized ultrapure water was prepared using an ultrapure-water purification system (Millipore, Bedford, MA, USA). Chemical standards were purchased from Sigma–Aldrich (St. Louis, MO, USA) and BioBioPha (Kunming, Yunnan, China).

### Sample Preparation for Metabolomics Analysis

Samples from the same batch of blueberry pastry filling samples prepared according to our previous study (8) were subjected to metabolomic analysis for the understanding of phytochemical changes under different processing procedures, including the raw filling, stir-fried, and baked samples (170°C, 23 min). All samples were lyophilized and crushed using a mixer mill (MM 400, Retsch, Haan, Germany) with a zirconia bead for 90 s at 30 Hz. Exactly 100 mg sample was then weighed accurately, extracted with 1 mL of 70% aqueous methanol overnight at 4°C by vigorously vortexing for three times. After centrifugation at 10,000 × g for 10 min, the extracts were filtered (0.22 μm) into a vial for UHPLC-MS/MS analysis. The quality control (QC) samples were prepared in triplicate by evenly mixing all the sample extracts tested and injected every six sample injections to monitor the measure repeatability.

### UHPLC-MS/MS Analysis

Exactly 2 μL of sample were injected and analyzed using a Shim-pack UFLC SHIMADZU CBM30A system (Shimadzu Corporation, Kyoto, Japan) interfaced to an Applied Biosystems 6500 QTRAP mass spectrometer (AB Sciex, Foster, CA, USA). The sample was separated on a Waters ACQUITY UPLC HSS T3 C18 column (100 mm × 2.1 mm, particle size 1.8 μm) (Waters Corporation, Milford, MA, USA) by using water (0.04% acetic acid) as solvent A and acetonitrile (0.04% acetic acid) as solvent B. The eluting gradient programme was as follows: 0.0–11.0 min, 5%−95% B; 11.0–12.0 min, 95% B; 12.0–12.1 min, 95%−5% B; and 12.1–15.0 min, 5% B. The flow rate was 0.4 mL/min. The temperature of column oven was kept at 40°C during the analysis. The effluent was connected to an ESI-triple quadrupole-linear ion trap (QQQ-LIT) mass spectrometer equipped with an ESI Turbo Ion-Spray interface operating in both positive and negative ion modes and controlled by Analyst 1.6.3 software. The ESI source operation parameters were as follows: source temperature 500°C; ion spray voltage (IS), 5500 V; and ion source gas I (GSI)/gas II (GSII)/curtain gas (CUR), 55/60/25 psi. Instrument tuning and mass calibration were performed with 10 and 100 μmol/L polypropylene glycol solutions in QQQ and LIT modes, respectively. QQQ scans were acquired as multiple reaction monitoring (MRM) experiments with collision gas set to 5 psi. Declustering potential (DP) and collision energy (CE) for individual MRM transitions were obtained with further DP and CE optimisation. A specific set of MRM transitions was monitored for each period according to the chemicals eluted within this period.

### Qualification and Quantification of Chemicals

For qualification of the chemicals, the primary and secondary mass spectrometry data were analyzed using MVDB V2.0 Database (Metware Biotechnology Co., Ltd., Wuhan, China) and the public database of metabolite information. The mass error comparing with the database was limited within ±0.4 Da, and the error for retention time was allowed within ±0.1 min. The interference from isotope signals, duplicate signals of K^+^, Na^+^, and ${\text{NH}}_{4}^{+}$ ions, and duplicate signals of fragment ions derived from other larger molecules were excluded. Structural analysis of chemicals was conducted in reference to the existing mass spectrometry databases, such as MassBank (http://www.massbank.jp), KNAPSAcK (http://kanaya.naist.jp/KNApSAcK), HMDB (http://www.hmdb.ca), and METLIN (http://metlin.scripps.edu/index.php). The chemicals were quantified in the MRM mode using QQQ mass spectrometry. After chemical data from different samples were obtained, the peak areas of the mass spectra of all substances were integrated, and the mass spectrum peaks of the same chemical in different samples were subjected to integration correction.

### Statistical Analysis

All samples were analyzed in triplicate. To investigate the effect of different thermal processing procedures on the composition of chemicals, hierarchical cluster analysis (HCA), principal component analysis (PCA), partial least squares-discriminant analysis (PLS-DA), and univariate analysis (UVA) on different samples were conducted with the MetaboAnalyst 4.0 platform. The thresholds for screening differential chemicals were set as follows: fold change ≥ 2 or ≤ 0.5 and VIP ≥ 1.

## Results and Discussion

Our previous study (8) showed that the flavonoid content varied considerably during the thermal processing of blueberry-filled pastries, and the procedures of stir-frying and baking contributed to the different levels of the loss of anthocyanins and flavonol glycosides. However, the degradation products of these flavonoids and the variation of the other phytochemicals were left undetected. Pastry filling is a complex food mixture that contains many components other than flavonoids, and various reactions may occur in addition to the simple degradation of flavonoids. A holistic view on the phytochemical changes during the production of blueberry-filled pastries will help us evaluate the nutritional changes during thermal processing. The metabolomic approach has been largely applied to investigate the metabolite changes during plant development, such as fruit ripening and seed germination (13–15).

This work applied metabolomic analysis to investigate the phytochemical changes of blueberry-filled pastry products during different thermal processing stages. All the samples were lyophilized and analyzed on the basis of dry weight mass to eliminate the water deviation between the samples in different thermal processing procedures.

### Full List of Chemical Components in Raw and Thermal-Treated Samples

A total ion current chromatogram of QC sample (an even mixture of all the samples involved in this study) and a multi-peak detection plot of chemicals in the MRM mode of the same sample are illustrated in Supplementary Figure 1. The chemicals in the samples were qualified and quantified by mass spectrometry by using the MVDB V2.0 database. The appearance and abundance of different substances in various samples were evaluated by MRM analysis. As shown in Supplementary Figures 1C,D, each peak displaying a distinct color represents one compound in the sample analyzed.

A total of 630 compounds were identified in the current study in the blueberry fillings of pastries collected at different processed stages, including 148 flavonoids, 21 carbohydrates, 69 organic acids, 24 quinates and their derivatives, 19 benzoic acid derivatives, 17 coumarins, 29 hydroxycinnamoyl derivatives, 5 proanthocyanidins, 2 flavonolignans, 28 amino acids and 56 of their derivatives, 18 fatty acids, 16 glycerolipids, 35 glycerophospholipids, 4 nicotinic acid derivatives, 57 nucleotides and their derivatives, 16 phenolamides, 7 indole derivatives, 3 pyridine derivatives, 4 cholines, 7 alcohols and polyols, 7 alkaloids, 7 tryptamine derivatives, 3 terpenoids, and 28 other chemicals. Amongst them, the largest group was flavonoid, which consisted of 13 anthocyanins, 10 catechin derivatives, 17 flavanones, 37 flavones, 26 flavone C-glycosides, 38 flavonols, and 7 isoflavones. The name and molecular formula of compounds, their precursor and product ions for qualification and quantification with MRM, and the peak integration values of the compounds are all listed in Supplementary Table 1. To the best of our knowledge, this study was the first to utilize the targeted UHPLC-MS/MS-based metabolomic approach in analyzing the chemical variations in fruit fillings of bakery products under different thermal treatments. Such a large amount of the compositional information provides a comprehensive understanding of the chemical changes in berries commonly enriched with bioactive polyphenols when subjected to thermal processing.

### Difference in the Composition Amongst Raw, Stir-Fried, and Baked Blueberry Fillings

**Figure 1:**
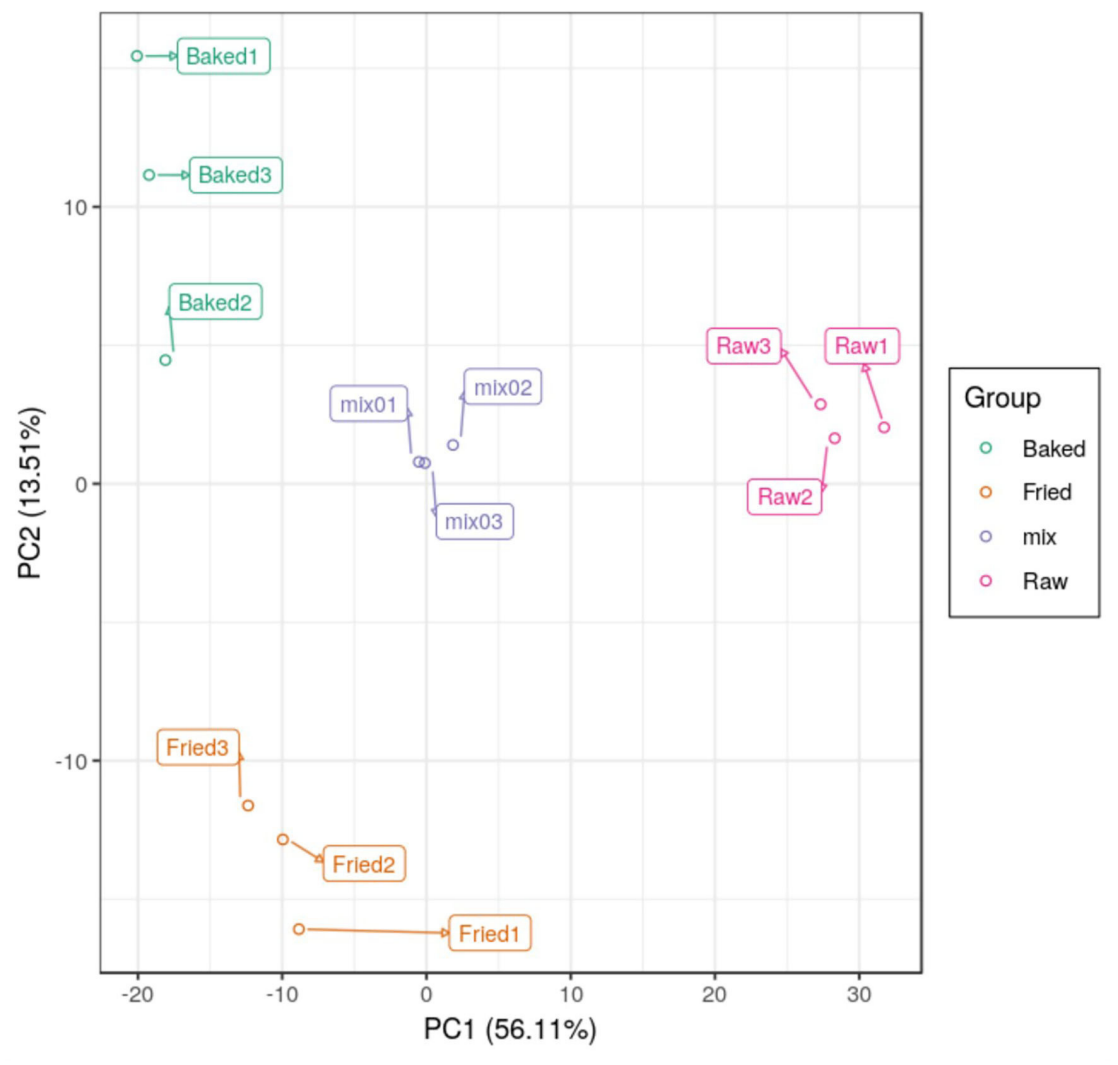
PCA scores scatter plot showing differences in composition between blueberry filling samples obtained at different thermal processing stages. mix, quality control sample; raw, fried, and baked refer to raw, fried, and baked fillings, respectively.

**Figure 2:**
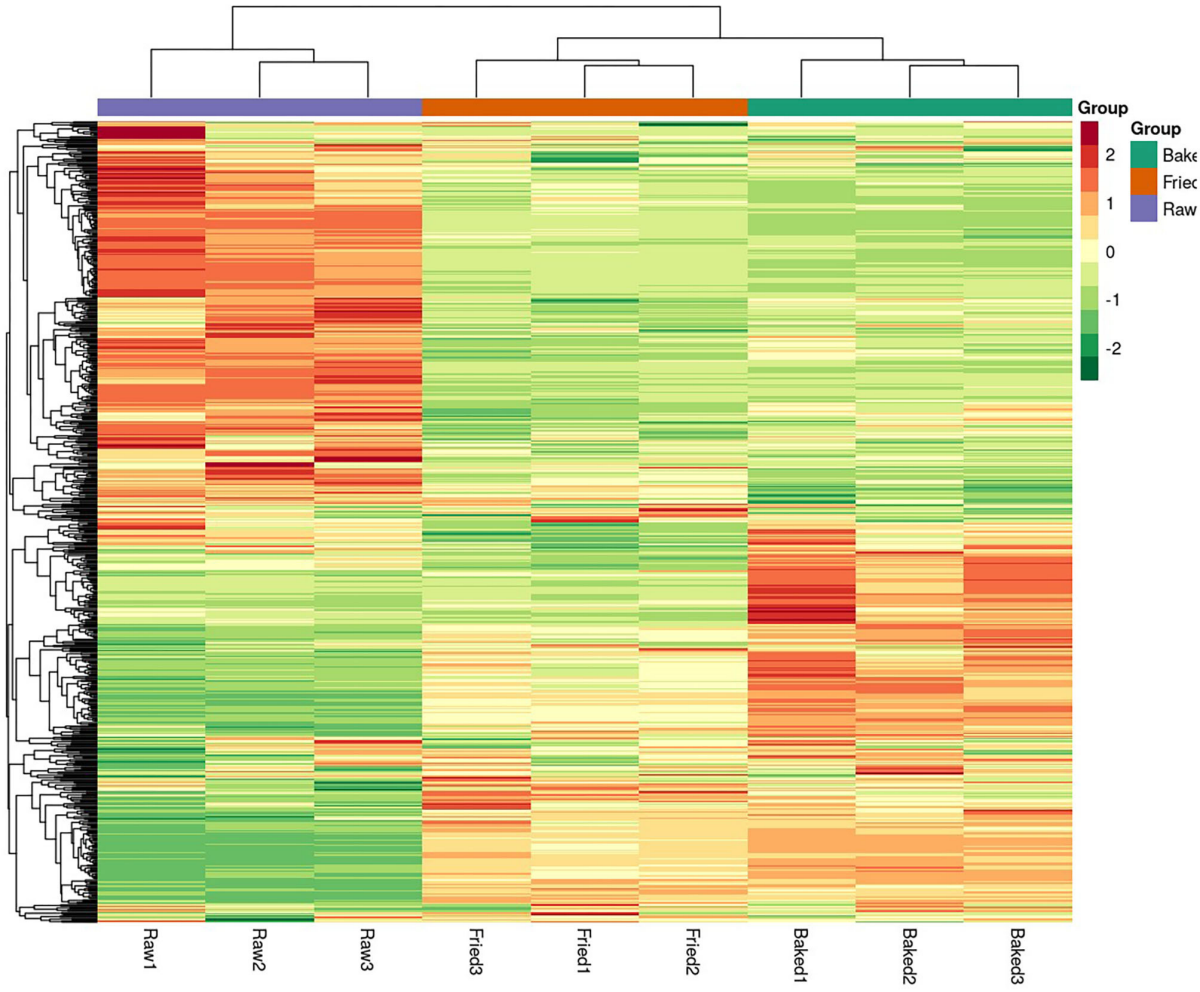
Heatmap of the hierarchical clustering analysis of differential chemicals amongst samples of raw, fried, and baked fillings. raw, fried, and baked refer to raw, fried, and baked fillings, respectively.

**Figure 3:**
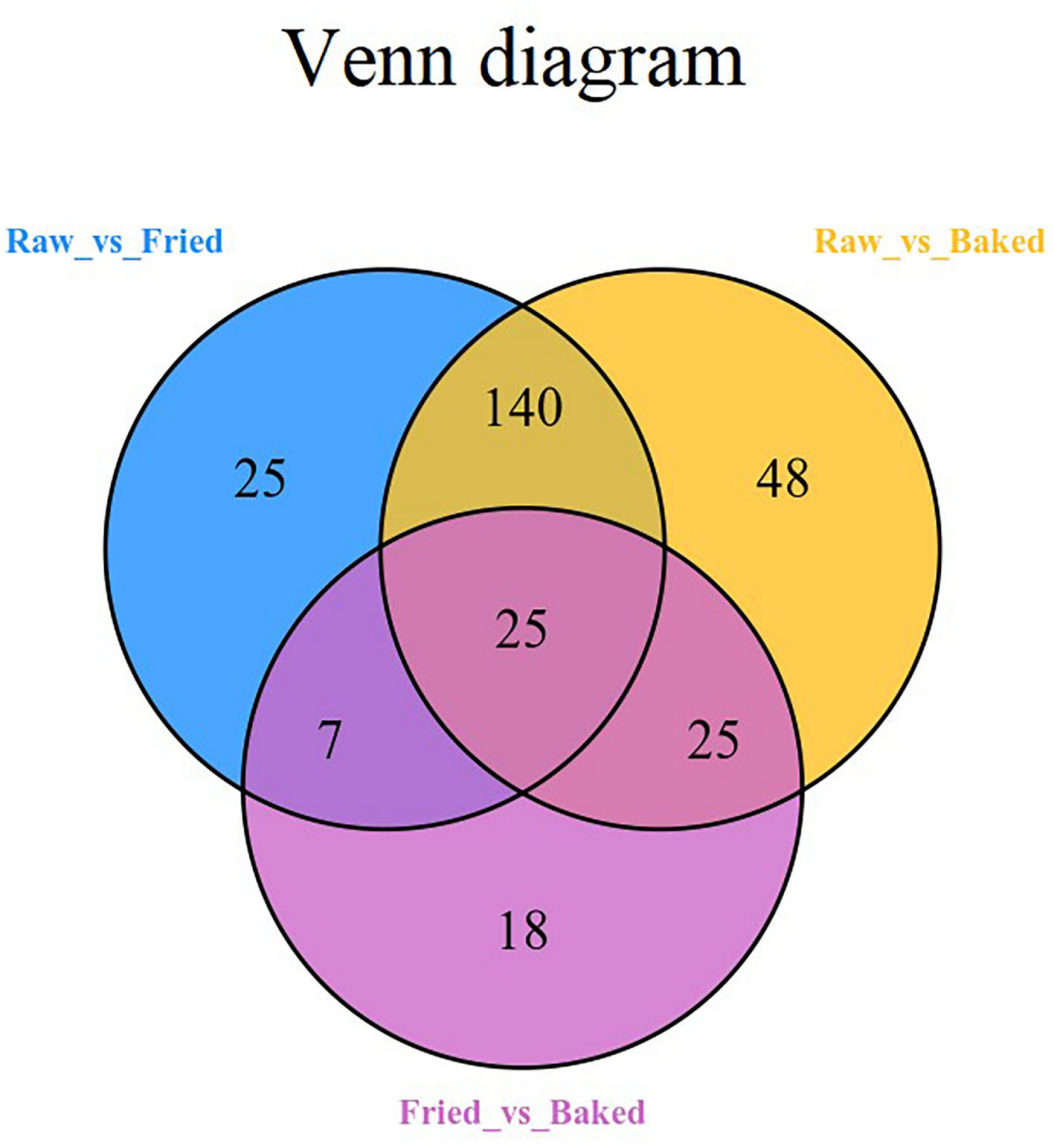
Venn diagram of the differential compounds of blueberry fillings at different processing stages. raw, fried, and baked refer to raw, fried, and baked fillings, respectively.

PCA and HCA were applied to analyze the data obtained for a full view of the differences between the blueberry filling samples obtained at different processing stages. The PCA score scatter plot (Figure 1) showed that samples obtained at different processing stages could be well-distinguished. The QC samples, as a mixture of all the three samples investigated, were located in the middle of the plot. The PC1 explained 56.11% of the variances of chemical data of all the samples investigated and predominantly distinguished the raw filling samples from the thermally treated samples, such as stir-fried and baked samples. PC2 explained only 13.51% of the variances of chemical data and distinguished mainly the stir-fried filling samples from the baked filling samples. This finding indicated that stir-frying and baking dramatically changed the composition of blueberry fillings. This finding was further confirmed by the heatmap of the hierarchical clustering analysis of differential chemicals amongst samples of raw, fried, and baked fillings (Figure 2). A total of 288 differential chemicals were screened by UVA. The HCA of the 288 differential chemicals in the three samples showed clear grouping patterns. The color sequence from red to green indicates a decrease in chemical contents. The abscissa of the thermogram indicates the different sample groups, and the ordinate indicates the differential chemicals identified between groups. According to the Venn diagram shown in Figure 3, 197 and 238 chemicals significantly differed (fold change ≤ 0.5 or ≥ 2, and VIP ≥ 1) between raw and fried fillings and between raw and baked fillings, respectively. However, the number of differential chemicals in fried filling vs. baked filling was only 75, which confirmed our previous finding that stir-frying was the major step contributing to the most variation in chemicals during blueberry pastry preparation (8). Consistently, the heatmap of HCA showed larger differences in abundance between raw and fried fillings than those between baked and fried fillings (Figure 2).

Amongst 288 differential compounds, 117 belonged to the group of phenolic compounds (Table 1), including 18 flavones (out of 37 flavones detected), 13 anthocyanins (13), 21 flavonols (38), 12 flavone C-glycosides (26), 8 flavanones (17), 14 benzoic acid derivatives (19), 14 hydroxycinnamoyl derivatives (29), 4 catechin derivatives (10), 5 coumarins (17), 6 quinates and their derivatives (24), 1 isoflavone (7), and 1 proanthocyanidin (5). The other compounds (Table 2) included 34 nucleotides and their derivatives (57), 29 glycerophospholipids (35), 21 organic acids (69), 5 amino acids (28), 22 amino acid derivatives (56), 13 glycerolipids (16), 8 carbohydrates (21), 8 phenolamides (16), 6 fatty acids (18), 3 tryptamine derivatives (7), 3 indole derivatives (7), 2 alkaloids (7), 2 terpenoids (3), 2 nicotinic acid derivatives (4), 2 alcohols and polyols (7), 1 pyridine derivative (3), and 1 choline (4). However, the flavonolignans detected in the fillings did not vary during the processing of pastries.

**Table 1:** List of differential chemicals belonging to phenolic compounds and their variations under different thermal processing procedures.

**Chemical**	**Class**	**Fried filling vs. raw filling**				**Baked filling vs. fried filling**				**Baked filling vs. raw filling**			
		**Variation percentage (%)**	**VIP**	**Fold change**	**Up/down regulated**	**Variation percentage (%)**	**VIP**	**Fold change**	**Up/down regulated**	**Variation percentage (%)**	**VIP**	**Fold change**	**Up/down regulated**
Peonidin *O*-hexoside	Anthocyanins	−69.84%	1.20	0.30	Down				-	−83.68%	1.24	0.16	Down
Cyanidin 3-*O*-malonylhexoside	Anthocyanins				-	−66.66%	1.11	0.00	Down	−100.00%	1.24	0.00	Down
Rosinidin *O*-hexoside	Anthocyanins	−73.88%	1.15	0.26	Down				-	−80.74%	1.22	0.19	Down
Cyanidin 3-*O*-galactoside	Anthocyanins	−76.09%	1.20	0.24	Down	−54.24%	1.42	0.46	Down	−89.01%	1.22	0.11	Down
Peonidin	Anthocyanins	−52.49%	1.13	0.47	Down				-	−57.59%	1.22	0.42	Down
Cyanidin *O*-syringic acid	Anthocyanins	−80.69%	1.20	0.19	Down				-	−88.68%	1.23	0.11	Down
Delphinidin	Anthocyanins	−68.14%	1.18	0.32	Down				-				-
Malvidin 3-*O*-galactoside	Anthocyanins	−69.24%	1.20	0.31	Down	−51.86%	1.42	0.49	Down	−85.07%	1.22	0.15	Down
Malvidin 3-*O*-glucoside	Anthocyanins	−71.62%	1.21	0.28	Down				-	−84.92%	1.22	0.15	Down
Delphinidin 3-*O*-galactoside	Anthocyanins	−81.20%	1.20	0.19	Down	−53.80%	1.40	0.46	Down	−91.23%	1.22	0.09	Down
Cyanidin 3,5-*O*-diglucoside	Anthocyanins	−100.00%	1.21	0.00	Down				-	−100.00%	1.24	0.00	Down
Petunidin 3-*O*-galactoside	Anthocyanins	−70.94%	1.20	0.29	Down	−55.12%	1.43	0.45	Down	−86.81%	1.22	0.13	Down
Pelargonidin 3-*O*-beta-D-glucoside	Anthocyanins	−71.03%	1.20	0.29	Down				-	−81.46%	1.24	0.18	Down
p-Aminobenzoate	Benzoic acid derivatives				-				-	110.35%	1.15	2.10	Up
8-Methyl-2-oxo-4-phenyl-2H-chromen-7-yl 4-(hexyloxy)benzoate	Benzoic acid derivatives	−66.26%	1.16	0.34	Down				-	−68.53%	1.20	0.30	Down
Methyl gallate	Benzoic acid derivatives	240.94%	1.18	3.39	Up				-	244.30%	1.22	3.38	Up
Vanillin	Benzoic acid derivatives	154.28%	1.13	2.43	Up				-	189.27%	1.15	2.66	Up
Gallic acid	Benzoic acid derivatives	504.62%	1.20	6.05	Up				-	614.96%	1.24	7.11	Up
2,5-dihydroxybenzoic acid	Benzoic acid derivatives	383.65%	1.19	4.78	Up				-	528.36%	1.23	6.08	Up
4-Hydroxybenzaldehyde	Benzoic acid derivatives	157.09%	1.19	2.57	Up				-	274.27%	1.23	3.70	Up
2,3-Dihydroxybenzoic acid	Benzoic acid derivatives	438.59%	1.20	5.39	Up					584.63%	1.24	6.77	Up
4-Hydroxybenzoic acid	Benzoic acid derivatives				-					123.11%	1.20	2.18	Up
2-(Formylamino)benzoic acid	Benzoic acid derivatives	143.61%	1.16	2.39	Up					207.25%	1.20	2.95	Up
Anthranilic acid	Benzoic acid derivatives	167.46%	1.18	2.65	Up				-	166.59%	1.20	2.63	Up
Syringaldehyde	Benzoic acid derivatives	202.84%	1.20	3.03	Up				-	245.88%	1.24	3.46	Up
Vanillic acid	Benzoic acid derivatives	172.84%	1.17	2.69	Up				-	225.36%	1.21	3.13	Up
Syringic acid	Benzoic acid derivatives	854.07%	1.20	9.52	Up				-	1072.42%	1.24	11.58	Up
Gallocatechin-gallocatechin	Catechin derivatives				-	−100.00%	1.62	0.00	Down	−100.00%	1.24	0.00	Down
Epigallocatechin	Catechin derivatives	590.56%	1.20	6.85	Up				-	511.38%	1.24	6.09	Up
Protocatechuic acid	Catechin derivatives	429.20%	1.20	5.29	Up				-	575.14%	1.24	6.70	Up
Protocatechuic aldehyde	Catechin derivatives	378.86%	1.20	4.72	Up				-	439.77%	1.23	5.25	Up
*O*-Feruloyl 4-hydroxylcoumarin	Coumarins	−58.15%	1.18	0.41	Down				-	−60.44%	1.14	0.39	Down
6,7-dihydroxycoumarin 7-*O*-quinic acid	Coumarins	−48.02%	1.08	0.50	Down				-				-
7-Hydroxy-5-methoxycoumarin	Coumarins				-				-	205.85%	1.22	2.98	Up
6-Methoxy-7,8-dihydroxycoumarin	Coumarins	1319.84%	1.13	10.17	Up	126.94%	1.59	2.21	Up	3402.35%	1.20	22.46	Up
Scoparone	Coumarins				-	−66.62%	1.18	0.00	Down	−99.96%	1.24	0.00	Down
Naringenin *O*-malonylhexoside	Flavanone	−68.07%	1.14	0.32	Down				-	−72.48%	1.18	0.27	Down
4′-Hydroxy-5,7-dimethoxyflavanone	Flavanone	114.58%	1.12	2.12	Up				-				-
Naringenin	Flavanone	236.74%	1.17	3.29	Up				-	369.93%	1.22	4.57	Up
Phloretin	Flavanone				-				-	113.43%	1.20	2.14	Up
Eriodictyol	Flavanone				-				-	150.83%	1.23	2.50	Up
Naringenin chalcone	Flavanone	235.51%	1.16	3.30	Up				-	373.97%	1.22	4.63	Up
sakuranetin	Flavanone	+	1.02	28.50	Up				-	+	1.04	34.41	Up
3,5,7,4′-Tetrahydroxyflavan	Flavanone				-				-	−62.12%	1.17	0.38	Down
Selgin *O*-malonylhexoside	Flavone	−65.72%	1.19	0.34	Down				-	−70.87%	1.21	0.29	Down
Chrysin *O*-malonylhexoside	Flavone	−68.60%	1.17	0.32	Down				-				-
Spinacetin	Flavone	−53.97%	1.18	0.46	Down				-				-
Chrysoeriol 7-*O*-rutinoside	Flavone	−55.04%	1.19	0.45	Down				-	−49.31%	1.15	0.49	Down
Chrysoeriol *O*-hexosyl-O-hexoside	Flavone	−54.84%	1.17	0.45	Down				-	−63.23%	1.22	0.37	Down
Apigenin 7-*O*-glucoside	Flavone				-				-	−59.36%	1.01	0.31	Down
Tricin 5-*O*-hexosyl-*O*-hexoside	Flavone	+	1.21	79789.26	Up				-	+	1.24	156066.67	Up
Tricin *O*-rhamnoside	Flavone	−99.96%	1.20	0.00	Down				-				-
Tricin 5-*O*-rutinoside	Flavone	−85.13%	1.18	0.15	Down				-	−91.00%	1.23	0.09	Down
Tricin *O*-hexosyl-*O*-syringin alcohol	Flavone	+	1.21	1511.37	Up				-	+	1.24	1384.53	Up
Tricin	Flavone				-				-	119.60%	1.18	2.18	Up
Acacetin *O*-acetyl hexoside	Flavone				-	124.85%	1.45	2.10	Up				-
Chrysoeriol *O*-acetylhexoside	Flavone	−49.75%	1.10	0.47	Down				-				-
Apigenin 7-*O*-neohesperidoside	Flavone				-				-	135.33%	1.18	2.29	Up
Chrysoeriol	Flavone				-				-	174.73%	1.20	2.75	Up
Nobiletin	Flavone				-	−37.89%	1.10	0.49	Down	−76.47%	1.21	0.23	Down
Tangeretin	Flavone	−51.21%	1.01	0.50	Down				-	−67.96%	1.19	0.30	Down
Butin	Flavone	240.50%	1.16	3.31	Up				-	369.59%	1.21	4.53	Up
C-hexosyl-chrysoeriol *O*-hexoside	Flavone C-glycosides	−63.15%	1.20	0.37	Down				-	−69.53%	1.24	0.30	Down
Chrysoeriol 6-C-hexoside 8-C-hexoside-*O*-hexoside	Flavone C-glycosides				-				-	−48.82%	1.07	0.47	Down
6-C-hexosyl-hesperetin *O*-hexoside	Flavone C-glycosides	−63.02%	1.19	0.36	Down				-	−63.59%	1.14	0.37	Down
di-C,C-hexosyl-apigenin	Flavone C-glycosides	−57.05%	1.19	0.43	Down				-	−73.00%	1.24	0.27	Down
C-hexosyl-luteolin *O*-*p*-coumaroylhexoside	Flavone C-glycosides				-				-	−53.56%	1.07	0.46	Down
Chrysoeriol C-hexosyl-*O*-rhamnoside	Flavone C-glycosides	−75.75%	1.20	0.24	Down				-	−86.98%	1.24	0.13	Down
8-C-hexosyl-luteolin *O*-hexoside	Flavone C-glycosides	−65.15%	1.15	0.35	Down				-	−69.36%	1.20	0.30	Down
Luteolin 8-C-hexosyl-*O*-hexoside	Flavone C-glycosides	−52.23%	1.16	0.48	Down				-	−59.09%	1.22	0.41	Down
C-hexosyl-apigenin C-pentoside	Flavone C-glycosides	−55.05%	1.16	0.44	Down				-				-
8-C-hexosyl chrysoeriol *O*-hexoside	Flavone C-glycosides	+	1.21	7587.37	Up				-	+	1.24	11791.56	Up
Hesperetin C-hexoside *O*-hexoside	Flavone C-glycosides	12525.71%	1.20	122.18	Up				-	22757.50%	1.24	225.37	Up
Luteolin C-hexoside	Flavone C-glycosides				-				-	100.60%	1.07	2.03	Up
methylQuercetin *O*-hexoside	Flavonol	−65.77%	1.12	0.35	Down				-	−75.25%	1.20	0.24	Down
Quercetin-3,4′-O-diglucoside	Flavonol	−74.25%	1.19	0.25	Down				-	−74.92%	1.23	0.25	Down
Isorhamnetin *O*-hexoside	Flavonol	−53.44%	1.20	0.47	Down				-	−54.86%	1.24	0.45	Down
Isorhamnetin 5-*O*-hexoside	Flavonol	−53.71%	1.19	0.46	Down				-	−54.57%	1.22	0.45	Down
Quercetin 7-*O*-malonylhexosyl-hexoside	Flavonol	−50.51%	1.14	0.49	Down				-	−60.68%	1.16	0.38	Down
Kaempferol	Flavonol				-				-	260.46%	1.23	3.59	Up
Quercetin	Flavonol	250.40%	1.20	3.50	Up				-	457.89%	1.24	5.58	Up
Quercetin-3-arabinoside	Flavonol	−58.34%	1.20	0.42	Down				-	−62.80%	1.24	0.37	Down
Kaempferol 3-*O*-rutinoside	Flavonol				-				-	−55.41%	1.22	0.44	Down
Myricetin	Flavonol				-				-	132.31%	1.23	2.30	Up
Dihydroquercetin	Flavonol	141.38%	1.05	2.32	Up				-	177.36%	1.14	2.56	Up
Isorhamnetin	Flavonol	379.09%	1.18	4.83	Up				-	809.31%	1.24	9.08	Up
Kaempferol 3-*O*-robinobioside	Flavonol				-				-	−52.56%	1.23	0.47	Down
Kaempferol 3,7-dirhamnoside	Flavonol				-				-	−55.36%	1.13	0.43	Down
Kaempferol 3-*O*-galactoside	Flavonol	−60.27%	1.19	0.40	Down				-	−63.19%	1.24	0.37	Down
7-*O*-methxyl quercetin	Flavonol	+	1.07	40.97	Up				-	+	1.13	77.36	Up
Syringetin	Flavonol	865.90%	1.19	9.65	Up				-	1433.02%	1.24	14.75	Up
Laricitrin	Flavonol	291.91%	1.19	3.93	Up				-	524.32%	1.23	6.16	Up
Quercetin 7-*O*-β-D-Glucuronide	Flavonol	−69.41%	1.12	0.31	Down				-	−72.95%	1.21	0.27	Down
Kaempferol-3-*O*-robinoside-7-*O*-rhamnoside	Flavonol	−62.14%	1.18	0.38	Down				-	−62.38%	1.16	0.37	Down
Morin	Flavonol	255.78%	1.18	3.58	Up				-	461.44%	1.24	5.59	Up
6-Hydroxymethylherniarin	Hydroxycinnamoyl derivatives				-				-	−50.96%	1.23	0.49	Down
Hydroxy-methoxycinnamate	Hydroxycinnamoyl derivatives				-				-	−52.88%	1.24	0.47	Down
4-Methoxycinnamic acid	Hydroxycinnamoyl derivatives	−71.75%	1.15	0.28	Down				-	−71.58%	1.19	0.28	Down
Cinnamic acid	Hydroxycinnamoyl derivatives	132.86%	1.11	2.32	Up				-	239.69%	1.19	3.28	Up
Ferulic acid	Hydroxycinnamoyl derivatives	138.88%	1.18	2.38	Up				-	207.47%	1.23	3.05	Up
Homovanillic acid	Hydroxycinnamoyl derivatives	−49.78%	1.13	0.49	Down				-				-
3-Hydroxy-4-methoxycinnamic acid	Hydroxycinnamoyl derivatives	148.04%	1.17	2.48	Up				-	211.79%	1.23	3.08	Up
p-Coumaric acid	Hydroxycinnamoyl derivatives	136.18%	1.15	2.34	Up				-	201.03%	1.21	2.91	Up
Sinapic acid	Hydroxycinnamoyl derivatives	1014.76%	1.20	11.11	Up				-	1407.51%	1.24	15.04	Up
Sinapyl alcohol	Hydroxycinnamoyl derivatives	128.98%	1.09	2.18	Up				-				-
p-Coumaryl alcohol	Hydroxycinnamoyl derivatives	−99.95%	1.20	0.00	Down	+	1.08	129.93	Up				-
Sinapinaldehyde	Hydroxycinnamoyl derivatives	186.15%	1.11	2.74	Up				-	259.93%	1.15	3.47	Up
p-Coumaraldehyde	Hydroxycinnamoyl derivatives	−53.49%	1.16	0.45	Down				-	−55.49%	1.20	0.43	Down
Caffeyl alcohol	Hydroxycinnamoyl derivatives	−54.80%	1.10	0.44	Down				-				-
Glycitin	Isoflavone	−49.66%	1.16	0.50	Down				-	−49.56%	1.09	0.49	Down
Procyanidin A3	Proanthocyanidins	256.20%	1.19	3.51	Up				-	335.10%	1.23	4.24	Up
1-*O*-Feruloyl quinic acid	Quinate and its derivatives	−56.96%	1.19	0.43	Down				-	−59.22%	1.23	0.40	Down
*O*-Feruloyl quinic acid	Quinate and its derivatives	136.07%	1.20	2.36	Up				-	153.40%	1.23	2.53	Up
5-*O*-*p*-Coumaroyl shikimic acid	Quinate and its derivatives	−64.98%	1.18	0.34	Down				-	−70.22%	1.19	0.30	Down
Eudesmoyl quinic acid	Quinate and its derivatives	359.65%	1.09	3.80	Up				-	388.12%	1.10	3.71	Up
5-*O*-*p*-coumaroyl shikimic acid *O*-hexoside	Quinate and its derivatives	−75.15%	1.20	0.25	Down				-	−86.58%	1.23	0.13	Down
Chlorogenic acid methyl ester	Quinate and its derivatives	−51.93%	1.13	0.47	Down				-	−57.72%	1.14	0.39	Down

*+, Newly occurred after the corresponding thermal treatment*.

**Table 2:** List of differential chemicals other than phenolic compounds and their variations under different thermal processing procedures.

**Chemical**	**Class**	**Fried filling vs. raw filling**				**Baked filling vs. fried filling**				**Baked filling vs. raw filling**			
		**Variation percentage (%)**	**VIP**	**Fold change**	**Up/Down regulated**	**Variation percentage (%)**	**VIP**	**Fold change**	**Up/Down regulated**	**Variation percentage (%)**	**VIP**	**Fold change**	**Up/Down regulated**
D(-)-Threose	Carbohydrates	−88.98%	1.20	0.11	Down				-	−86.79%	1.23	0.13	Down
Ribulose-5-phosphate	Carbohydrates	122.78%	1.02	2.01	Up				-				-
Glucosamine	Carbohydrates				-				-	+	1.01	55.75	Up
D(+)-Melezitose O-rhamnoside	Carbohydrates	−48.78%	1.15	0.50	Down	−58.26%	1.58	0.41	Down	−79.40%	1.22	0.21	Down
Trehalose 6-phosphate	Carbohydrates				-				-	235.19%	1.16	3.37	Up
D(+)-Melezitose	Carbohydrates				-				-	236.92%	1.03	2.56	Up
D-(+)-Glucono-1,5-lactone	Carbohydrates	122.72%	1.20	2.22	Up				-	149.99%	1.22	2.50	Up
L-Fucose	Carbohydrates	543.66%	1.19	6.45	Up				-	392.67%	1.20	4.91	Up
L-Histidine	Amino acids				-				-	−59.43%	1.20	0.40	Down
L-(+)-Arginine	Amino acids				-				-	−59.68%	1.23	0.40	Down
L-Methionine	Amino acids	−65.76%	1.19	0.34	Down				-	−64.68%	1.24	0.35	Down
L-Glutamine	Amino acids	−64.62%	1.20	0.35	Down	−56.97%	1.58	0.42	Down	−85.10%	1.24	0.15	Down
L(+)-Ornithine	Amino acids	−52.14%	1.17	0.48	Down				-				-
Lysine butyrate	Amino acid derivatives				-	+	1.34	60.90	Up	+	1.13	213.85	Up
N-Acetylmethionine	Amino acid derivatives	−74.42%	1.20	0.26	Down				-	−78.16%	1.23	0.22	Down
Acetyl tryptophan	Amino acid derivatives				-				-	158.90%	1.03	2.53	Up
L-Glutamine O-hexside	Amino acid derivatives	329.84%	1.19	4.28	Up	−51.52%	1.56	0.48	Down	104.88%	1.23	2.04	Up
L-Glutamic acid O-glucoside	Amino acid derivatives	277.32%	1.17	3.65	Up				-	156.72%	1.21	2.50	Up
3-(2-Naphthyl)-D-alanine	Amino acid derivatives				-	−53.37%	1.43	0.44	Down	−53.56%	1.01	0.42	Down
Glutathione oxidized	Amino acid derivatives				-				-	−57.12%	1.23	0.43	Down
N-Acetyl-L-glutamic acid	Amino acid derivatives	139.85%	1.19	2.38	Up				-	143.20%	1.22	2.41	Up
(-)-3-(3,4-Dihydroxyphenyl)-2-methylalanine	Amino acid derivatives	201.64%	1.17	2.89	Up				-	183.83%	1.18	2.67	Up
Pyrrole-2-carboxylic acid	Amino acid derivatives	666.89%	1.20	7.55	Up				-	879.38%	1.23	9.62	Up
Phenylacetyl-L-glutamine	Amino acid derivatives				-				-	197.73%	1.10	2.71	Up
N-(3-Indolylacetyl)-L-alanine	Amino acid derivatives				-				-	−48.02%	1.11	0.50	Down
Glutathione reduced form	Amino acid derivatives	−66.52%	1.12	0.30	Down				-	−81.85%	1.21	0.17	Down
N′-Formylkynurenine	Amino acid derivatives				-				-	106.42%	1.22	2.07	Up
L-Pipecolic acid	Amino acid derivatives	148.41%	1.20	2.47	Up				-	191.76%	1.24	2.90	Up
3-N-Methyl-L-histidine	Amino acid derivatives	−68.42%	1.18	0.31	Down				-	−53.13%	1.15	0.45	Down
Phe-Phe	Amino acid derivatives				-				-	−49.19%	1.13	0.49	Down
S-(5′-Adenosyl)-L-methionine	Amino acid derivatives	−99.31%	1.04	0.01	Down	−66.43%	1.18	0.01	Down	−100.00%	1.24	0.00	Down
N-Phenylacetylglycine	Amino acid derivatives				-	436.94%	1.59	5.48	Up	257.74%	1.18	3.65	Up
Hexanoyl glycine	Amino acid derivatives				-				-	208.35%	1.02	2.59	Up
CYS-GLY	Amino acid derivatives				-	+	1.11	2.37	Up	−61.97%	1.13	0.36	Down
H-HomoArg-OH	Amino acid derivatives				-				-	−52.09%	1.18	0.47	Down
1,4-dihydro-1-Methyl-4-oxo-3-pyridinecarboxamide	Pyridine derivatives	−56.87%	1.19	0.43	Down				-				-
Pantothenol	Alcohols and polyols	−85.25%	1.20	0.15	Down				-	−88.42%	1.24	0.12	Down
1,5-Anhydro-D-glucitol	Alcohols and polyols	1059.15%	1.20	11.23	Up				-	956.15%	1.23	9.96	Up
sn-Glycero-3-phosphocholine	Cholines				-				-	223.75%	1.21	3.17	Up
N′, N″-disinapoylspermidine	Phenolamides				-				-	−59.34%	1.17	0.40	Down
N-Feruloyl spermidine	Phenolamides	−65.26%	1.17	0.34	Down	−47.97%	1.34	0.49	Down	−82.69%	1.18	0.17	Down
Spermidine	Phenolamides	1080.60%	1.19	11.07	Up				-	616.06%	1.21	6.89	Up
Agmatine	Phenolamides	390.76%	1.19	4.72	Up				-	315.40%	1.08	3.80	Up
N-(4′-O-glycosyl)-p-coumaroyl agmatine	Phenolamides	−59.81%	1.19	0.40	Down				-	−75.60%	1.24	0.24	Down
N′-Feruloyl putrescine	Phenolamides				-				-	−99.99%	1.24	0.00	Down
N-Feruloyl putrescine	Phenolamides	−65.83%	1.09	0.34	Down				-	−80.08%	1.21	0.20	Down
N-Acetylputrescine	Phenolamides	633.73%	1.19	7.16	Up				-	589.41%	1.20	6.84	Up
N2-methylguanosine	Nucleotide and its derivatives				-				-	−56.92%	1.21	0.43	Down
Adenosine 3′-monophosphate	Nucleotide and its derivatives	354.12%	1.19	4.54	Up				-	384.82%	1.23	4.79	Up
Nicotinic acid adenine dinucleotide	Nucleotide and its derivatives	−79.78%	1.20	0.20	Down	−58.38%	1.51	0.42	Down	−91.49%	1.23	0.08	Down
Inosine 5′-monophosphate	Nucleotide and its derivatives	307.27%	1.18	4.01	Up				-	302.25%	1.20	3.86	Up
iP7G	Nucleotide and its derivatives	454.83%	1.19	5.41	Up				-	959.51%	1.23	10.60	Up
Adenosine 5′-monophosphate	Nucleotide and its derivatives	260.29%	1.19	3.59	Up				-	280.91%	1.22	3.77	Up
Guanosine 5′-monophosphate	Nucleotide and its derivatives				-				-	150.43%	1.15	2.51	Up
Uridine 5′-diphospho-D-glucose	Nucleotide and its derivatives	−63.26%	1.17	0.36	Down	−50.79%	1.54	0.49	Down	−82.24%	1.24	0.18	Down
2′-Deoxyinosine-5′-monophosphate	Nucleotide and its derivatives	−65.70%	1.20	0.34	Down				-	−80.44%	1.22	0.19	Down
Adenosine O-ribose	Nucleotide and its derivatives	−58.63%	1.20	0.41	Down				-	−52.89%	1.16	0.47	Down
Uridine	Nucleotide and its derivatives	−58.53%	1.19	0.41	Down				-				-
Cytosine	Nucleotide and its derivatives				-	140.29%	1.57	2.36	Up				-
Adenine	Nucleotide and its derivatives	−50.73%	1.19	0.49	Down				-				-
β-Nicotinamide mononucleotide	Nucleotide and its derivatives	−84.25%	1.20	0.16	Down	−58.14%	1.58	0.40	Down	−93.66%	1.24	0.06	Down
1-Methylxanthine	Nucleotide and its derivatives	+	1.21	5315.81	Up				-	+	1.24	4970.37	Up
Adenosine	Nucleotide and its derivatives	107.54%	1.20	2.07	Up				-	158.06%	1.10	2.60	Up
5-Methyluridine	Nucleotide and its derivatives	+	1.21	18098.52	Up				-	+	1.24	20249.26	Up
Guanine	Nucleotide and its derivatives				-				-	102.46%	1.22	2.01	Up
Inosine	Nucleotide and its derivatives				-	108.31%	1.54	2.03	Up				-
Guanosine	Nucleotide and its derivates	−55.49%	1.20	0.45	Down				-				-
Deoxyguanosine	Nucleotide and its derivates	−77.80%	1.19	0.22	Down	677.30%	1.57	7.20	Up				-
2′-Deoxycytidine-5′-monophosphate	Nucleotide and its derivates	+	1.21	40852.96	Up				-				-
1-Methyladenosine	Nucleotide and its derivates	854.79%	1.20	9.43	Up				-	1488.22%	1.24	15.92	Up
5′-Deoxy-5′-(methylthio)adenosine	Nucleotide and its derivates	276.54%	1.20	3.77	Up				-	242.45%	1.23	3.43	Up
Guanosine monophosphate	Nucleotide and its derivates	317.07%	1.19	4.07	Up				-	419.44%	1.23	5.07	Up
1,7-Dimethylxanthine	Nucleotide and its derivates	−96.78%	1.20	0.03	Down	163.97%	1.41	2.37	Up	−92.39%	1.23	0.08	Down
Cytidine 5′-monophosphate	Nucleotide and its derivates	123.40%	1.18	2.22	Up				-	230.48%	1.21	3.27	Up
Cyclic AMP	Nucleotide and its derivates	−99.98%	1.21	0.00	Down	+	1.63	38850.37	Up	503.61%	1.14	6.28	Up
8-Hydroxy-2-deoxyguanosine	Nucleotide and its derivates				-	188.52%	1.24	2.96	Up				-
Cytidine	Nucleotide and its derivates				-	155.64%	1.55	2.58	Up	103.59%	1.13	2.02	Up
Guanosine 3′,5′-cyclic monophosphate	Nucleotide and its derivates	2206.16%	1.20	22.35	Up				-	3051.38%	1.24	29.88	Up
Deoxyadenosine	Nucleotide and its derivates				-	309.47%	1.48	4.07	Up	598.04%	1.16	7.21	Up
2-(dimethylamino)guanosine	Nucleotide and its derivates				-	98.90%	1.31	2.02	Up				-
Hypoxanthine-9-β-D-arabinofuranoside	Nucleotide and its derivates				-	108.11%	1.50	2.08	Up				-
N-hexosyl-p-coumaroyl serotonin	Tryptamine derivatives	−64.78%	1.19	0.35	Down				-	−76.01%	1.21	0.24	Down
N-Feruloyl serotonin	Tryptamine derivatives	−54.01%	1.05	0.41	Down				-				-
N-Feruloyl tryptamine	Tryptamine derivatives	−65.74%	1.20	0.34	Down				-	−61.59%	1.23	0.38	Down
Camptothecin	Alkaloids	115.81%	1.14	2.09	Up				-	150.83%	1.15	2.37	Up
Betaine	Alkaloids	211.70%	1.20	3.09	Up	602.30%	1.61	7.03	Up	2121.08%	1.24	21.72	Up
Phytocassane D	Terpenoids	−71.27%	1.18	0.28	Down				-	−65.36%	1.09	0.36	Down
Limonin	Terpenoids	+	1.21	1666.70	Up				-	+	1.24	2001.59	Up
Nicotinate ribonucleoside	Nicotinic acid derivatives				-				-	−56.92%	1.23	0.43	Down
Nicotinic acid	Nicotinic acid derivatives	117.40%	1.20	2.17	Up				-	170.28%	1.23	2.70	Up
5-methoxyindole-3-carbaldehyde	Indole derivatives	−71.95%	1.13	0.27	Down				-				-
Indole-5-carboxylic acid	Indole derivatives				-				-	163.20%	1.16	2.52	Up
5-Hydroxyindole-3-acetic acid	Indole derivatives				-				-	−56.11%	1.23	0.44	Down
Methylglutaric acid	Organic acids	−65.52%	1.19	0.34	Down	−51.92%	1.51	0.47	Down	−83.88%	1.23	0.16	Down
2-Aminoethanesulfinic acid	Organic acids	470.39%	1.14	4.89	Up				-	695.50%	1.18	6.59	Up
2–Furoic acid	Organic acids	+	1.21	841940.74	Up				-	+	1.24	1084903.70	Up
Glutaric acid	Organic acids	133.63%	1.20	2.33	Up				-	166.27%	1.24	2.66	Up
4-Oxopentanoate	Organic acids	214.66%	1.03	2.79	Up				-	168.42%	1.11	2.41	Up
Terephthalic acid	Organic acids				-				-	136.19%	1.19	2.33	Up
2-Picolinic acid	Organic acids	208.13%	1.20	3.08	Up				-	309.17%	1.24	4.09	Up
Homogentisic acid	Organic acids	1160.72%	1.20	12.56	Up				-	1485.63%	1.24	15.76	Up
D-Pantothenic acid	Organic acids	−65.31%	1.18	0.34	Down				-				-
3-Hydroxy-3-methyl butyric acid	Organic acids	130.04%	1.16	2.24	Up				-	137.70%	1.20	2.33	Up
D-Erythronolactone	Organic acids	185.54%	1.17	2.78	Up	106.46%	1.52	2.06	Up	475.33%	1.22	5.74	Up
Creatine	Organic acids				-	1146.83%	1.61	12.45	Up	678.39%	1.21	7.80	Up
2-Aminoethanesulfonic acid	Organic acids	578.57%	1.20	6.73	Up				-	1188.64%	1.23	12.97	Up
Suberic acid	Organic acids	197.14%	1.14	2.85	Up				-	177.00%	1.18	2.65	Up
Citraconic acid	Organic acids	+	1.21	231022.22	Up				-	+	1.24	256744.44	Up
Mandelic acid	Organic acids				-				-	117.32%	1.06	2.20	Up
A-Ketoglutaric acid	Organic acids	114.87%	1.20	2.15	Up				-	126.45%	1.22	2.26	Up
3,4-Dihydroxybenzeneacetic acid	Organic acids	943.16%	1.19	9.73	Up				-	1275.36%	1.23	12.81	Up
ethylmalonate	Organic acids	106.55%	1.14	2.00	Up				-	144.32%	1.19	2.42	Up
(Rs)-Mevalonic acid	Organic acids	1509.63%	1.18	14.18	Up				-	2493.85%	1.23	22.74	Up
trans,trans-Muconic acid	Organic acids	261.50%	1.18	3.50	Up				-	473.77%	1.23	5.59	Up
LysoPC 16:1	Lipids_Glycerophospholipids	160.83%	1.13	2.45	Up				-	206.03%	1.20	2.94	Up
LysoPC 18:2	Lipids_Glycerophospholipids	202.86%	1.14	2.93	Up	122.13%	1.42	2.13	Up	538.39%	1.22	6.24	Up
LysoPC 18:3	Lipids_Glycerophospholipids	236.76%	1.04	3.03	Up	123.49%	1.19	2.11	Up	578.88%	1.18	6.40	Up
LysoPC 16:0	Lipids_Glycerophospholipids	196.70%	1.11	2.86	Up				-	406.37%	1.20	5.03	Up
LysoPC 18:1 (2n isomer)	Lipids_Glycerophospholipids				-	192.53%	1.52	2.85	Up	192.96%	1.20	2.90	Up
LysoPC 18:3 (2n isomer)	Lipids_Glycerophospholipids				-				-	122.54%	1.16	2.13	Up
LysoPC 14:0	Lipids_Glycerophospholipids				-	341.02%	1.57	4.35	Up	201.51%	1.16	2.92	Up
LysoPE 18:2 (2n isomer)	Lipids_Glycerophospholipids				-				-	253.95%	1.18	3.46	Up
LysoPE 18:0 (2n isomer)	Lipids_Glycerophospholipids	226.78%	1.03	2.84	Up	220.96%	1.43	2.96	Up	765.64%	1.19	8.42	Up
LysoPE 18:2	Lipids_Glycerophospholipids	−52.50%	1.16	0.47	Down	165.92%	1.45	2.64	Up				-
LysoPC 18:1	Lipids_Glycerophospholipids				-				-	280.22%	1.15	3.58	Up
LysoPE 18:0	Lipids_Glycerophospholipids	−91.76%	1.16	0.07	Down	1511.34%	1.59	15.50	Up				-
LysoPC 10:0	Lipids_Glycerophospholipids	−55.99%	1.05	0.40	Down	332.05%	1.50	3.97	Up				-
LysoPC 19:0	Lipids_Glycerophospholipids	187.22%	1.11	2.79	Up	597.01%	1.59	6.54	Up	1900.05%	1.23	18.22	Up
LysoPC 15:1	Lipids_Glycerophospholipids				-	222.20%	1.52	3.09	Up	232.09%	1.18	3.23	Up
LysoPC 15:0	Lipids_Glycerophospholipids				-	275.37%	1.56	3.77	Up	217.58%	1.16	3.14	Up
PC 19:2/16:0	Lipids_Glycerophospholipids				-	431.85%	1.25	3.50	Up	59007.31%	1.07	58.69	Up
LysoPC 18:0 (2n isomer)	Lipids_Glycerophospholipids				-	197.08%	1.47	2.98	Up	181.91%	1.12	2.71	Up
LysoPC 17:0	Lipids_Glycerophospholipids				-	165.57%	1.38	2.72	Up				-
LysoPE 18:1	Lipids_Glycerophospholipids	−80.13%	1.17	0.20	Down	134.47%	1.52	2.23	Up	−54.06%	1.16	0.45	Down
LysoPE 18:1 (2n isomer)	Lipids_Glycerophospholipids	−62.34%	1.17	0.37	Down	291.54%	1.61	3.80	Up				-
LysoPC 20:4	Lipids_Glycerophospholipids				-	232.49%	1.50	3.27	Up	238.49%	1.17	3.24	Up
LysoPC 14:0 (2n isomer)	Lipids_Glycerophospholipids	−53.71%	1.12	0.46	Down	269.23%	1.56	3.48	Up				-
LysoPC 16:0 (2n isomer)	Lipids_Glycerophospholipids	295.77%	1.15	3.82	Up	127.99%	1.38	2.18	Up	745.08%	1.22	8.32	Up
LysoPC 18:0	Lipids_Glycerophospholipids				-	401.07%	1.56	5.04	Up	521.49%	1.18	5.80	Up
LysoPC 20:1 (2n isomer)	Lipids_Glycerophospholipids				-	128.93%	1.53	2.30	Up				-
LysoPC 20:1	Lipids_Glycerophospholipids				-	129.41%	1.53	2.31	Up				-
LysoPE 14:0 (2n isomer)	Lipids_Glycerophospholipids				-	139.83%	1.55	2.26	Up				-
LysoPE 16:0 (2n isomer)	Lipids_Glycerophospholipids	185.01%	1.10	2.77	Up				-	419.60%	1.22	5.19	Up
DGMG (18:2) isomer1	Lipids_Glycerolipids				-	192.32%	1.12	3.02	Up				-
DGMG (18:2) isomer3	Lipids_Glycerolipids				-	420.39%	1.48	5.14	Up	683.96%	1.18	7.44	Up
MAG (18:2) isomer1	Lipids_Glycerolipids				-	440.62%	1.47	5.60	Up	597.46%	1.15	6.26	Up
MAG (18:4) isomer2	Lipids_Glycerolipids				-	157.95%	1.46	2.48	Up				-
MAG (18:1) isomer2	Lipids_Glycerolipids				-	191.58%	1.18	2.97	Up				-
MAG (18:2)	Lipids_Glycerolipids	−56.04%	1.08	0.40	Down				-				-
MAG (18:3) isomer3	Lipids_Glycerolipids				-	201.68%	1.13	2.87	Up	320.09%	1.03	4.11	Up
MGMG (18:2) isomer1	Lipids_Glycerolipids				-	994.36%	1.60	11.05	Up	1361.25%	1.23	14.08	Up
MAG (18:3) isomer4	Lipids_Glycerolipids	−50.80%	1.04	0.44	Down				-				-
MAG (18:3) isomer2	Lipids_Glycerolipids				-	−77.99%	1.20	0.23	Down				-
MAG (18:1) isomer1	Lipids_Glycerolipids	−70.79%	1.17	0.28	Down				-				-
MGMG (18:2) isomer2	Lipids_Glycerolipids				-	219.11%	1.29	2.64	Up	207.38%	1.11	2.91	Up
MAG (18:3) isomer1	Lipids_Glycerolipids				-	121.22%	1.12	2.23	Up				-
14,15-Dehydrocrepenynic acid	Lipids_Fatty acids	−93.89%	1.20	0.06	Down				-	−92.86%	1.24	0.07	Down
delta-Tridecalactone	Lipids_Fatty acids	+	1.21	5796.89	Up				-	+	1.24	6310.04	Up
Punicic acid	Lipids_Fatty acids	−76.28%	1.16	0.23	Down				-	−67.90%	1.18	0.32	Down
9,10-EODE	Lipids_Fatty acids	−71.04%	1.09	0.30	Down				-	−56.12%	1.18	0.43	Down
9-HOTrE	Lipids_Fatty acids	−78.29%	1.15	0.22	Down				-	−75.34%	1.22	0.24	Down
12,13-EODE	Lipids_Fatty acids				-	145.94%	1.43	2.15	Up				-
DIMBOA glucoside	Others				-	524.36%	1.58	6.18	Up	567.10%	1.19	6.33	Up
2-Aminoethylphosphonate	Others				-				-	−57.49%	1.21	0.42	Down
Aminopurine	Others	105.95%	1.20	2.06	Up				-	196.03%	1.22	2.97	Up
4-Methyl-5-thiazoleethanol	Others	458.52%	1.20	5.54	Up				-	557.25%	1.24	6.52	Up
D-erythro-Dihydrosphingosine	Others				-	214.58%	1.40	2.73	Up				-
Phellodensin F	Others	−69.62%	1.17	0.31	Down	−55.57%	1.51	0.44	Down	−86.65%	1.23	0.13	Down
Cocamidopropyl betaine	Others	−51.36%	1.12	0.48	Down				-				-
L-Carnitine	Others				-	589.72%	1.59	6.94	Up				-
NADP	Others	−84.28%	1.19	0.16	Down				-	−92.34%	1.22	0.08	Down

*+, Newly occurred after the corresponding thermal treatment(s)*.

### Variation in the Phenolic Composition of Blueberry Fillings During Thermal Processing

All the anthocyanins showed decreasing trends during the preparation of blueberry-filled pastries, and the most variation occurred at the stir-frying stage. This result was in accordance with our previous finding (8) in which anthocyanins were the most sensitive toward stir-frying and less sensitive to baking, which should be most likely attributed to the higher temperature and longer period of heating occurred during stir-firing than baking. Moreover, the low concentration of oxygen in the sealed fillings might decrease the reduction in flavonoids under baking conditions (16), while abundant air perfused into the fillings and accelerated the degradation of flavonoids during stir-frying.

Except for cyanidin 3-*O*-malonylhexoside, the contents of all the anthocyanins decreased by 52–100% during stir-frying in the current study. However, during baking, cyanidin 3-*O*-malonylhexoside along with four other anthocyanins, namely cyanidin 3-*O*-galactoside, malvidin 3-*O*-galactoside, delphinidin 3-*O*-galactoside, and petunidin 3-*O*-galactoside, decreased by 52–67%. Cyanidin 3,5-*O*-diglucoside was neither detected in the fried filling nor in the baked filling. Therefore, it was totally degraded after thermal treatment. Cyanidin 3,5-*O*-diglucoside was identified by Hou et al. (17) in the anthocyanin-rich extract from black rice and was found to be comparable to the other anthocyanins detected in terms of thermal sensitivity because of the low values of activation energies (*E*_*a*_). Cevallos-Casals and Cisneros-Zevallos (18) suggested that sugars and their degradation products might accelerate the degradation of anthocyanins because of the association of the degradation rate of anthocyanin with the degradation rate of sugars to furfural-type compounds derived from the Maillard reaction. 2-Furoic acid, which is an oxidation product of furfural that originated from the decomposition of sugars, and ascorbic acid (19, 20) was found to be produced in the fillings after thermal processing (Table 1). Moreover, Louarme and Billaud (21) demonstrated that the production of 2-furoic acid depended on the oxidative degradation reactions of ascorbic acid rather than the thermal degradations, which might explain the remarkable occurrence of the compound during stir-frying (high oxygen exposure and long heating period) but mild changes under baking (low oxygen exposure in the sealed fillings and short period of heating).

Some flavonol aglycones detected in the current study increased considerably after thermal treatment by stir-frying, accompanying the decreases in the contents of their glycosides (Table 1). For example, the abundance of quercetin increased by 2.5 times, whereas some of its glycosides, such as quercetin-3,4′-*O*-diglucoside, quercetin 7-*O*-malonylhexosyl-hexoside, and quercetin 3-arabinoside, decreased remarkably after stir-frying. Kaempferol glycosides, such as kaempferol 3-*O-*galactoside, kaempferol 3-*O*-rutinoside, kaempferol 3-*O*-robinobioside, kaempferol 3,7-dirhamnoside, and kaempferol-3-*O*-robinoside-7-*O*-rhamnoside, decreased after thermal processing, whereas the aglycone kaempferol increased. The decreasing trends of the flavonol glycosides could be attributed to the deglycosylation that occurred during thermal processing as discussed in our previous study (8).

Changes in other phenolic compounds in blueberry filling during pastry preparation can be also determined by the widely targeted metabolomic analysis with UHPLC-MS/MS. For example, protocatechuic acid in the blueberry filling increased by four times after stir-frying. Protocatechuic acid was reported to be thermally degraded from cyanidin by the liberation of the catecholic B-ring or from flavonoids such as quercetin (9). It shared the same ortho-dihydroxyphenyl chemical moiety with cyanidin, which was reported to be critical for the biological properties of anthocyanins (22, 23). Thus, the degradation of cyanidin to protocatechuic acid might not result in the reduction of bioactivity. Protocatechuic acid was demonstrated to possess chemopreventive activity against several different types of cancers in animal and cell studies (24, 25). Coumarins are another group of phenolic compounds that possess various biological and therapeutic properties such as anti-oxidant, anti-microbial, anti-viral, anti-diabetic, anti-coagulant, estrogenic, vasodilator, anti-convulsant, anti-inflammatory, anti-hypertensive, and anti-cancer activities (26). In blueberry fillings, the amount of 6-methoxy-7,8-dihydroxycoumarin increased remarkably by 13 times after stir-frying, and by 34 times by the end of thermal processing. The degradation of anthocyanins might contribute to the increase (27). Moreover, 4-hydroxybenzoic acid was also reported to be the thermal degradation product of anthocyanin pelargonidin-3-glucoside (28). Although its content did not increase significantly in the filling after stir-frying, the abundance of pelargonidin-3-glucoside decreased remarkably. However, the final abundance of the compound increased significantly in the baked product compared with the raw filling. Other benzoic acid derivatives, such as gallic acid, vanillin, syringaldehyde, methyl gallate, 2,5-dihydroxybenzoic acid, 4-hydroxybenzaldehyde, anthranilic acid, and 2,3-dihydroxybenzoic acid, showed elevations in the filling after stir-frying. The increases in the amounts of vanillin, syringaldehyde, phenol, and phenol derivatives during heat treatment might be attributed to the thermal degradation of lignin (29). The thermal decomposition of the lignin polymer commonly started with the cleavage of the α-ether and β-ether bonds. As a result, a mixture of phenol-, guaiacyl-, and syringyl-type derivatives with their substituents in the aromatic ring was released (30); the attack of oxygen might lead to the formation of aromatic aldehydes and ketones (31). Amongst the hydroxycinnamoyl derivatives detected, 12 varied under the stir-frying treatment. Ferulic acid, cinnamic acid, *p*-coumaric acid, and sinapic acid showed increasing trends similar to that of benzoic acid derivatives. Moreover, the catechin derivatives, protocatechuic acid, protocatechuic aldehyde, and epigallocatechin showed increasing trends during stir-frying. The increases in these phenolic acids might be explained by the thermal degradation of flavonoids and the release of bound phenolic compounds from the insoluble polymers in the fruits under heat treatments (32, 33).

Amongst the flavanones, the abundance of naringenin and its chalcone increased after stir-frying, whereas that of naringenin *O*-malonylhexoside decreased. Lou et al. (34) observed increases in the amounts of naringenin, tangeretin, and gallic acid in the immature calamondin after heat treatment at 150°C for 1.5 h and attributed these increases to the liberation of the compound from the immature calamondin peel during heating. Heat treatment could cause the degradation of cell wall structure to release the bound phenolic acids. Accordingly, the contents of tangeretin and gallic acid in the blueberry filling also increased after stir-frying (Table 1). However, none of the flavanones displayed notable changes in fillings during baking. Interestingly, sakuranetin, which is a methylation product of naringenin and has not been detected in the raw fillings, was produced in fried and baked fillings. Although the biosynthesis of sakuranetin from naringenin in plants (35) and the reversable *O*-demethylation of sakuranetin to naringenin in living tissues under the presence of cytochrome P450 monooxygenases have been well-studied (36), the formation of these components and their interactions and correlations are difficult to speculate without further investigations.

Flavone C-glycosides displayed no variation during baking; during stir-frying, seven flavone C-glycosides showed significant decreases in abundance (Table 1). By contrast, two flavone C-glycosides, namely, 8-C-hexosyl chrysoeriol *O*-hexoside and hesperetin C-hexoside *O*-hexoside, increased significantly in the fillings during stir-frying. Moreover, 8-C-hexosyl chrysoeriol *O*-hexoside was only observed after stir-frying. Therefore, it is a novel degradation product that mostly originated from flavonoids. Amongst the flavones, tricin 5-*O*-hexosyl-*O*-hexoside and tricin *O*-hexosyl-*O*-syringin alcohol were novel degradation products that appeared after stir-frying. The other flavones either decreased or remained constant during stir-frying.

### Variation in Other Chemicals in Blueberry Fillings During Thermal Processing

Other than flavonoids, the other chemicals in the blueberry fillings were also fully investigated by the widely targeted metabolomic analysis with UHPLC-MS/MS to obtain a comprehensive view on the variation in whole chemical compounds in blueberry filling during pastry preparation and gain further insight into the underlying mechanisms.

During thermal processing, the abundance of sucrose decreased by 32% at the stage of stir-frying, while the amount of glucose remained unchanged after stir-frying. The contents of the oxidized products of glucose, gluconic acid and glucono-1,5-lactone, increased by 52 and 123%, respectively (Table 2). However, only the variation in glucono-1,5-lactone was statistically significant. The increases in the amount of gluconic acid and glucono-1,5-lactone might be attributed to the oxidation of glucose induced by H_2_O_2_ in presence of ferrous ion (37), in which H_2_O_2_ can be produced from ascorbic acid catalyzed by Cu(II) (38).

Amongst all the 28 amino acids detected, only l-methionine, l-glutamine, and l-(+)-ornithine decreased significantly in the blueberry filling after stir-frying. Baking only affected the amount of l-glutamine. In comparison with the raw filling, baked filling showed higher levels of l-histidine, l-(+)-arginine, l-methionine, and l-glutamine. Pyrrole-2-carboxylic acid, which is a product of the Maillard browning reaction between xylose and amino acid/peptides, increased sharply by 667% after stir-frying (39, 40).

Fang et al. (41) found that triphosphates and diphosphates of nucleotides could be degraded into monophosphate analogs or even nucleosides during heating. This finding might explain the considerable increases in the amounts of nucleosides and their monophosphates in blueberry fillings after heating. The abundance of adenosine, adenosine 3′-monophosphate, inosine 5′-monophosphate, adenosine 5′-monophosphate, guanosine monophosphate, cytidine 5′-monophosphate, and guanosine 3′,5′-cyclic monophosphate increased by 108, 354, 307, 260, 317, 123, and 2206%, respectively, after stir-frying. 2′-Deoxycytidine-5′-monophosphate appeared as a new product in the filling after stir-frying. Surprisingly, the degradation products of methylated nucleosides extensively increased; for instance, 1-methyladenosine increased by 855%, and 5-methyluridine and 1-methylxanthine were produced only after the thermal treatment of stir-frying. The results demonstrated that nucleosides and even free nucleotides would be degraded during heat treatment (41). Moreover, the thermal processing, especially at acidic conditions, might even elicit DNA degradation. Bitskinashvili et al. (42) reported that the combined thermal-acid treatment at 100°C and pH 2–4 could cause considerable degradations of maize and wheat DNA.

Amongst the seven alkaloids, only two showed significant variation after thermal processing, and both displayed increasing trends. However, three tryptamine derivatives were detected to be significantly different and showed reducing trends under the thermal treatment of stir-frying. The other components, such as organic acids, lipids, and amino acid derivatives, showed individually different variation behaviors under thermal processing (Table 2). Amongst the organic acids, only methylglutaric and d-pantothenic acids decreased in terms of abundance upon thermal treatments. Citraconic acid and 2-furoic acid occurred as new products after the thermal treatment of stir-frying. Glutaric acid, which is a degradation product that originated from sugar and lignin under heat treatment, increased by 1.3 times in the filling after stir-frying. d-Erythronolactone, which is a lactone formed by the reaction of d-xylose and calcium hydroxide (43), increased by 186 and 106%, respectively, after stir-frying and baking. Butter was incorporated into the pastry fillings and exposed to high temperature during stir-frying. Heating of butter increased its lactone content (43). As a result, the tridecalactone, which is a component in butter, was observed after stir-frying.

## Conclusions

In this study, we applied a widely targeted metabolomic approach to investigate the global chemical changes during different thermal processing procedures, namely, stir-frying and baking, which were involved in the preparation of traditional blueberry filled pastry. A total of 630 chemicals were detected in blueberry fillings, and 288 were screened to be differential chemicals as samples underwent different thermal treatments. In comparison with baking, stir-frying contributed the most to the deviation of the abundance of components in blueberry fillings. Anthocyanin was the most sensitive toward thermal treatments. The other classes of phytochemicals, such as glycerophospholipids, nucleotides and their derivatives, benzoic acid derivatives, flavonols, flavones, flavanones, flavone C-glycosides, hydroxycinnamoyl derivatives, and phenolamides, were also comparably sensitive toward thermal processing. The variation in the chemicals and the novel compounds that appeared after thermal processing detected by this widely targeted metabolomic analysis could provide important insights into the degradation mechanism of phytochemicals and interactions between food ingredients. However, metabolomic analysis for the total understanding of the variation in chemical features and underlying mechanisms during food preparation and corresponding reactions remains a major challenge. The currently developed metabolomic databases are mostly specific to food products, such as FooDB, which is a subset of the Human Metabolite Database (44). This database contains information about thousands of phytochemicals but lacks information on the corresponding chemical degradation products or interaction products. Additional efforts should be applied on the development of metabolomic approaches and their application in investigating food processing.

## Data Availability Statement

The original contributions presented in the study are included in the article/Supplementary Material, further inquiries can be directed to the corresponding author/s.

## Author Contributions

JZ, WH, SO, and PL contributed conception and design of the study. JZ, ZW, NY, and KZ performed the experiments. JZ, ZW, and PL organized the database and performed the statistical analysis. JZ and SO wrote the first draft of the manuscript. PL and ZW revised the manuscript. All authors contributed to the article and approved the submitted version.

## Conflict of Interest

The authors declare that the research was conducted in the absence of any commercial or financial relationships that could be construed as a potential conflict of interest.
