# Supplementary material for: Widely Targeted UHPLC-MS/MS Metabolomic Analysis on the Chemical Variation in Blueberry-Filled Pastries During Processing
Source: Front Nutr. 2020 Nov 9;7:569172. doi: 10.3389/fnut.2020.569172 (PMC7680857; doi:10.3389/fnut.2020.569172)
Supplement: Supplementary file 1 [file Table_1.DOCX]

Supplementary Material

# Supplementary Figures and Tables

## Supplementary Figures


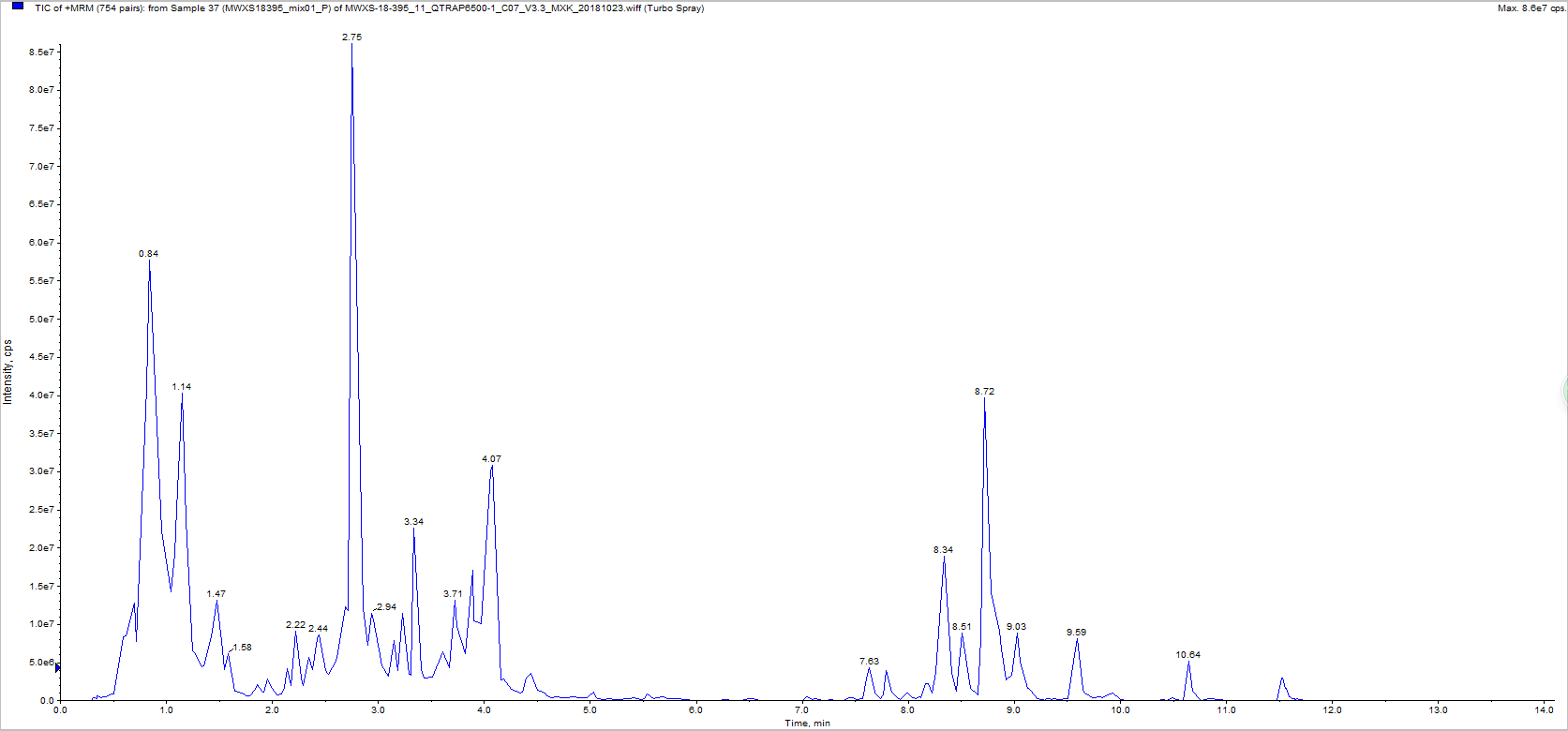


**A**


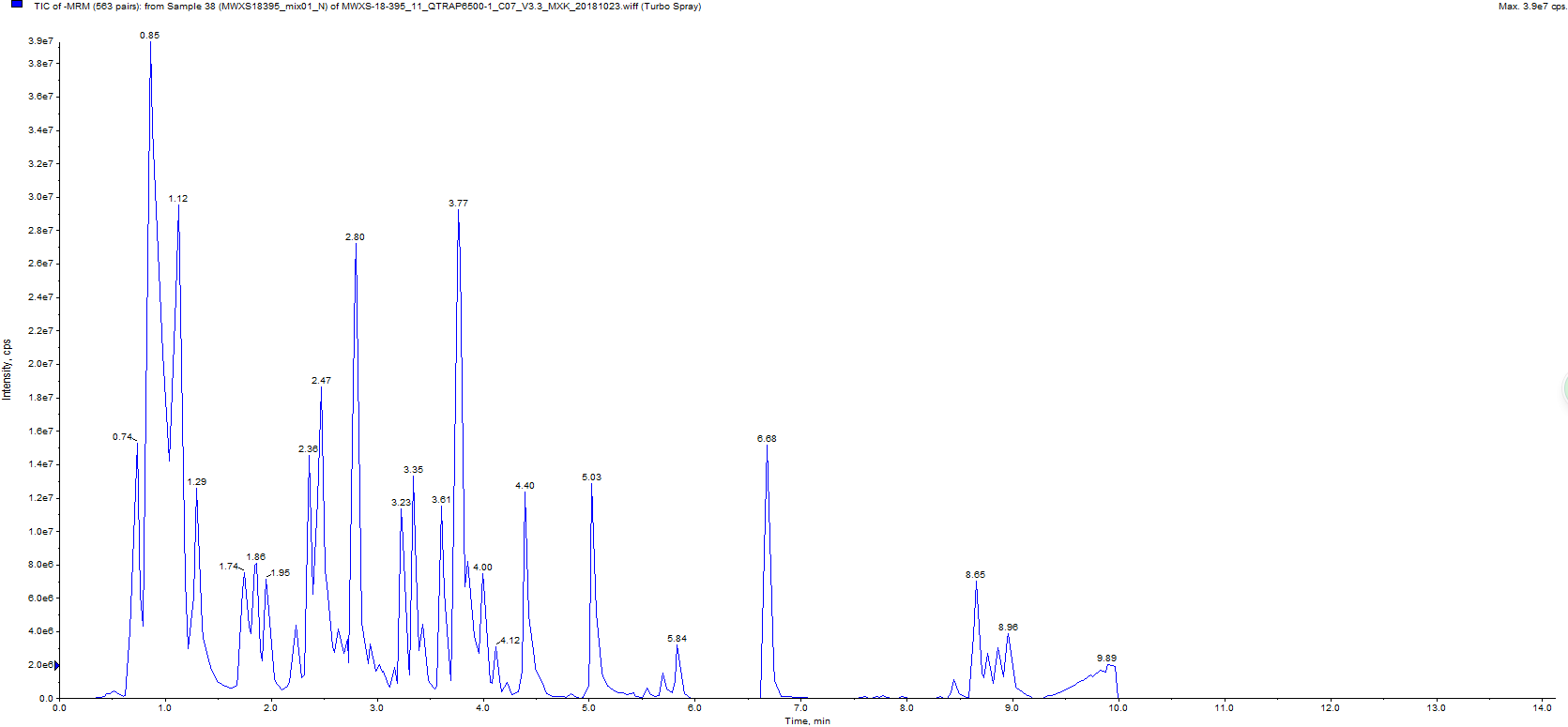


**B**


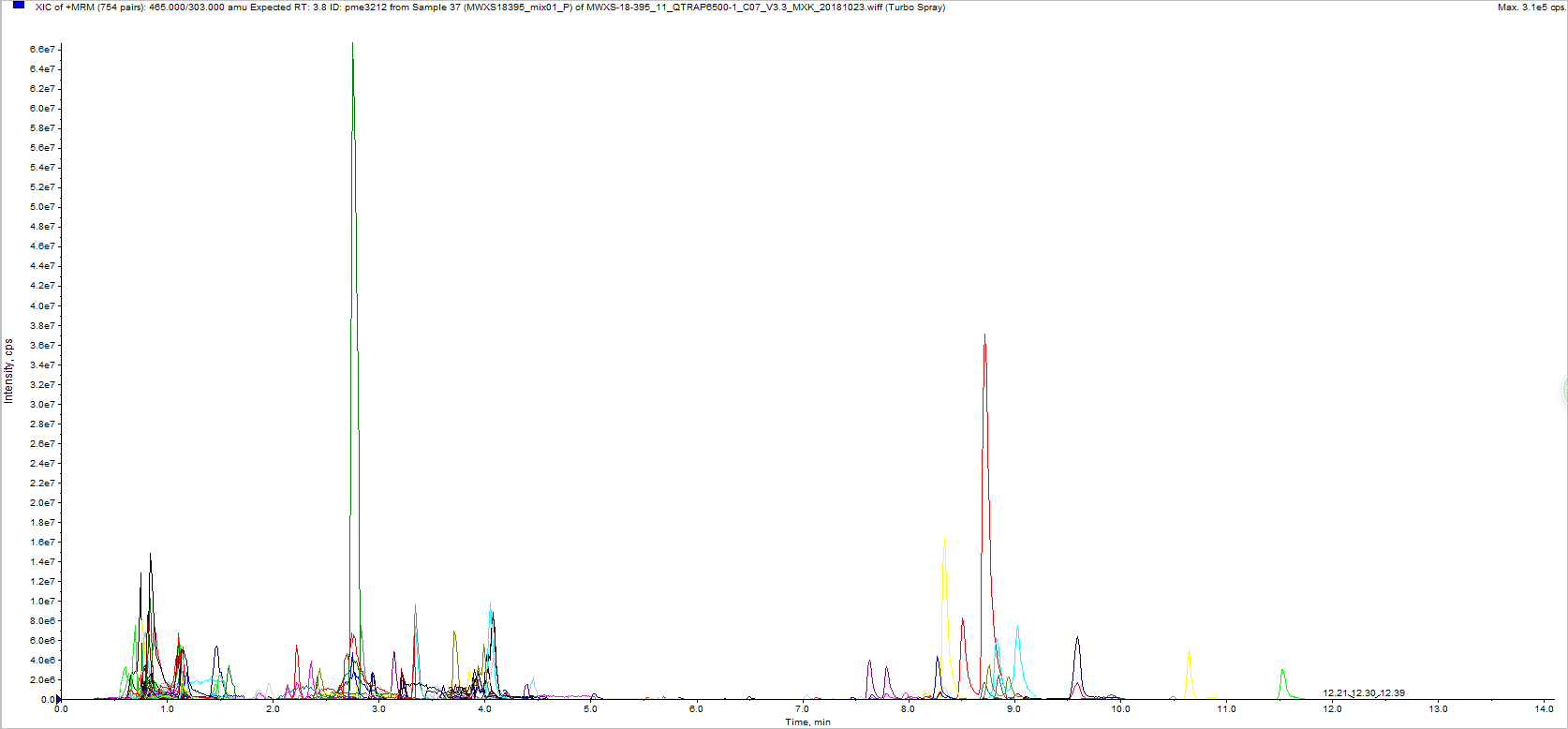


**C**


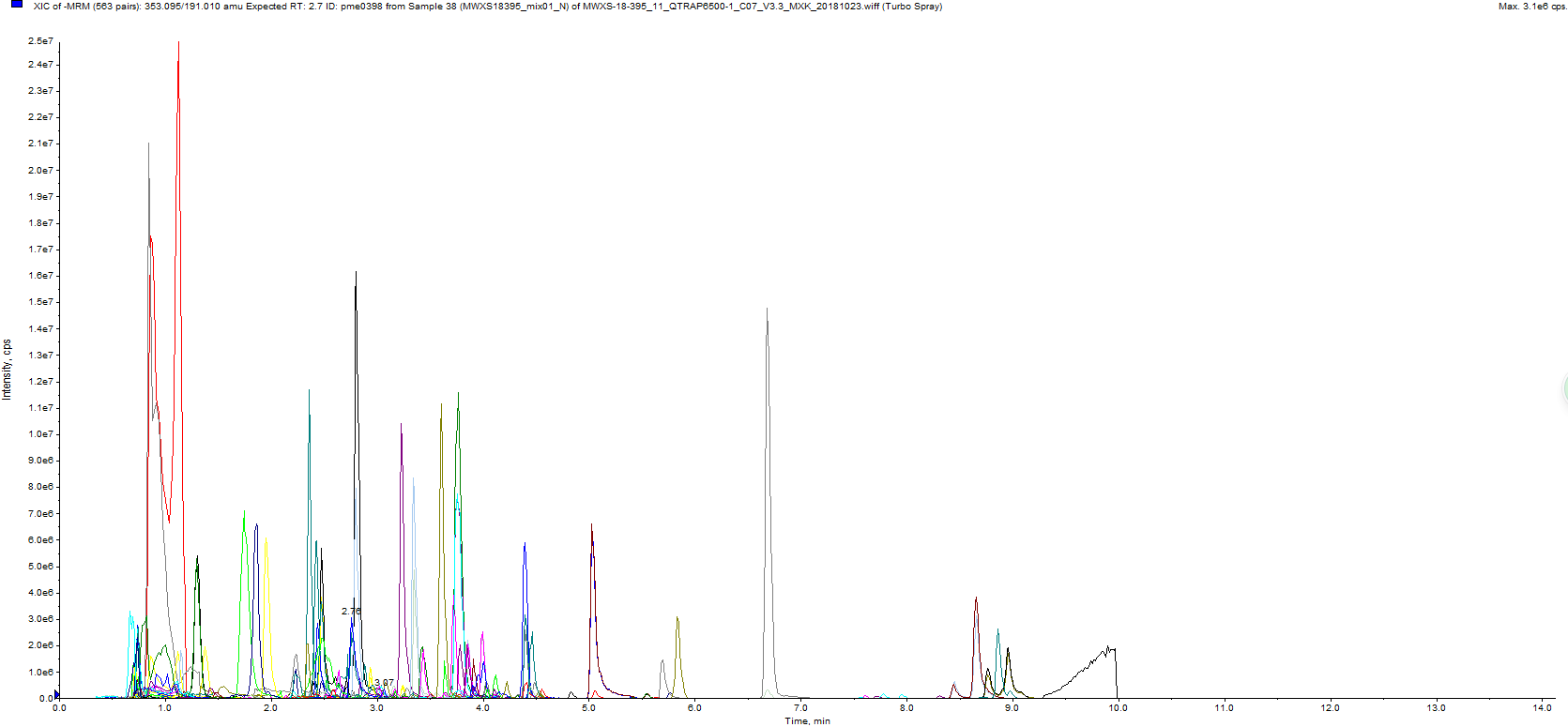


**D**

**Supplementary Figure S1.** Total ion current (TIC) chromatogram of a quality control (QC) sample by mass spectrometry detection (A and B for positive mode and negative mode, respectively) and the multi-peak detection plot of metabolites of the same sample in the multiple reaction monitoring (MRM) mode (C and D for positive mode and negative mode, respectively).

## Supplementary Tables

**Supplementary** **Table S1** (uploaded as an excel file)**.** Data for the qualification and quantification of the compounds by mass spectrometric analyses.
